# The Prosecution of Quacks

**Published:** 1898-09-03

**Authors:** 


### The Prosecution of Quacks.

A paragraph, manifestly inspired from some unknown source, has lately appeared in identical terms in several newspapers, setting forth that the "medical authorities" are about to prosecute with increased stringency their "campaign against unqualified medical men." The paragraph also contained a statement that "the General Medical Council has been appealed to as to whether it would be an offence for a qualified medical man to assist *in the administration of anæsthetics by an unqualified dental practitioner*." "The reply," we are told, "allows no doubt whatever upon the subject." The Registrar of the Council declared that not only was it an offence, but that the Dental Committee would recommend the Council to closely investigate every case of the kind reported to it. The above statement, still according to the paragraph, "means that administration under such circumstances will be declared to be infamous conduct—the strongest step ever taken in this direction." The *Globe*, in some editorial comments upon this precious piece of composition, approves of the intended activity of the "authorities" against the unqualified man, but thinks it "a strong order" to declare that a doctor who administers an anæsthetic for an unqualified dentist is guilty of infamous conduct. It seems to the *Globe* that the General Medical Council has sufficient work before it "in discouraging the obvious quack, instead of issuing such a tremendous fulmination against such a very small sin."

We have quoted all this nonsense as a convenient illustration of the extraordinary want of knowledge of the laws affecting the medical profession, and of the work and duties of the Medical Council, that prevails even in quarters in which some glimmering of knowledge on these matters might not unreasonably be expected. We do not think there is any other profession concerning which half a dozen newspapers would have accepted for insertion a paragraph containing nearly as many blunders as words, and would have demonstrated their good faith in doing so by commenting upon the blunders as if they were verities. There can be very little doubt that the paragraph in question is the composition of some person or society having "an axe to grind," and that our contemporaries have fallen into a cleverly laid snare from which a very moderate amount of knowledge of medical legislation and practice would have protected them.

What really happened is, that last March the council of the Dundee branch of the British Medical Association asked the opinion of the Medical Council as to whether a warning issued by the Council concerning the "covering" of unqualified practitioners by qualified men would extend to the administration of anæsthetics by the latter to the patients of unqualified persons who were transgressing the law by calling themselves dentists. The Executive Committee of the Council expressed an opinion that the practice described was "most reprehensible," and referred the application to the Council itself. The Council on the 31st of last May referred the letter for further report to the members of its Dental Committee, and will probably receive their report at its November meeting. So far, the Council has made no declaration on the subject, and it has always most carefully avoided laying down any general definition of infamous conduct. Its powers are limited to pronouncing the conduct of a particular person to be "infamous in a professional respect," and it only does this after full investigation of the charge, and of the evidence by which it is supported. It is very likely that, if a medical practitioner were charged with "covering," by habitually administering anæsthetics for a so-called but unqualified dentist, and with thus assisting him in the habitual perpetration of a fraud upon the public, and if the charge were proved, that the Council might pronounce the conduct to be infamous, and might deprive the offender of his place on the Medical Register. No such case has yet been heard, and no decision can possibly have been arrived at. In the meanwhile, the Council has no more to do with "discouraging the obvious quack" than has the editor of the *Globe* himself. That gentleman, or the Medical Council, or any other individual or corporation, could put the law in force against anyone who transgressed it by describing himself as a medical practitioner when he was not so in reality. The "obvious quack," as a rule, takes care to violate no law, although he sometimes comes into collision with the Apothecaries Act of 1815, which, if he be sharp, he soon learns how to evade. According to high legal opinion the powers and duties of the Council have no relation to quacks or quackery, and are only designed to protect the public by securing that registered members of the medical profession are competent and respectable persons.

